# Dynamics in Redox-Active Molecules Following Ischemic Preconditioning in the Brain

**DOI:** 10.3390/neurolint16030040

**Published:** 2024-05-09

**Authors:** Terezia Lysikova, Anna Tomascova, Maria Kovalska, Jan Lehotsky, Katarina Leskova Majdova, Peter Kaplan, Zuzana Tatarkova

**Affiliations:** 1 Department of Medical Biochemistry, Jessenius Faculty of Medicine in Martin, Comenius University in Bratislava, 03601 Martin, Slovakia; pesulova1@uniba.sk (T.L.); ann.benova@gmail.com (A.T.); jan.lehotsky@uniba.sk (J.L.); majdova6@uniba.sk (K.L.M.); peter.kaplan@uniba.sk (P.K.); 2 Department of Histology and Embryology, Jessenius Faculty of Medicine in Martin, Comenius University in Bratislava, 03601 Martin, Slovakia; maria.kovalska@uniba.sk

**Keywords:** oxidative stress, ischemic preconditioning, redox-active proteins, mitochondria, neuroprotection

## Abstract

It is well known that the brain is quite vulnerable to oxidative stress, initiating neuronal loss after ischemia-reperfusion (IR) injury. A potent protective mechanism is ischemic preconditioning (IPC), where proteins are among the primary targets. This study explores redox-active proteins’ role in preserving energy supply. Adult rats were divided into the control, IR, and IPC groups. Protein profiling was conducted to identify modified proteins and then verified through activity assays, immunoblot, and immunohistochemical analyses. IPC protected cortex mitochondria, as evidenced by a 2.26-fold increase in superoxide dismutase (SOD) activity. Additionally, stable core subunits of respiratory chain complexes ensured sufficient energy production, supported by a 16.6% increase in ATP synthase activity. In hippocampal cells, IPC led to the downregulation of energy-related dehydrogenases, while a significantly higher level of peroxiredoxin 6 (PRX6) was observed. Notably, IPC significantly enhanced glutathione reductase activity to provide sufficient glutathione to maintain PRX6 function. Astrocytes may mobilize PRX6 to protect neurons during initial ischemic events, by decreased PRX6 positivity in astrocytes, accompanied by an increase in neurons following both IR injury and IPC. Maintained redox signaling via astrocyte-neuron communication triggers IPC’s protective state. The partnership among PRX6, SOD, and glutathione reductase appears essential in safeguarding and stabilizing the hippocampus.

## 1. Introduction

The brain serves as the orchestrator of organ function and the regulator of whole-body metabolism, yet it possesses minimal energy reserves. This inherent limitation imposes a substantial burden on the organism, underscoring the necessity for a precise interconnection between energy metabolism and neurotransmission [1]. The energy metabolism in the brain is characterized as a complex system involving metabolites and enzymatic/transport mechanisms [2]. Distinct metabolic processes delineate each type of nerve cell, necessitating a continual exchange of metabolites that serve as both energy precursors and paracrine signals. An example of this metabolic interplay is evident in astrocyte–neuron collaboration, where the glycolytic metabolism of astrocytes contrasts with the mitochondrial oxidative activity of neurons. This connection facilitates the transport of glycolytically derived metabolites, such as L-lactate and L-serine, from astrocytes to neurons. These metabolites play a crucial role in meeting energy requirements for the maintenance of cell homeostasis, redox status, and modulation of neurotransmitter–receptor interactions [3].

Mitochondria, intracellular organelles primarily responsible for maintaining energy homeostasis, display dynamic behavior. Recent studies have furnished evidence indicating the continuous movement, fusion, division, and interaction of mitochondria, thus exerting a substantial influence on cellular degeneration and plasticity in neural cells. Beyond ATP production mediated through oxidative phosphorylation, mitochondrial functions encompass the regulation of neuronal Ca^2+^ homeostasis [4], apoptosis [5], or necroptosis [6] as well as participation in cellular signaling, repair, and maintenance [7,8]. These physiological processes, specifically the energy requirements for neurotransmission and synaptic plasticity, lead to the elevation of reactive oxygen species (ROS) levels within neurons [9,10]. The increased formation of ROS disrupts the endogenous redox equilibrium, resulting in oxidative stress, cellular death, and consequential tissue damage [11]. The brain, with its high and specific metabolic activity, is extremely susceptible to the pathological effects caused by oxidative stress events. Various factors, such as higher oxygen demand, a large amount of easily oxidizable lipids, and increased iron levels, can collectively act as prooxidants [12]. This is particularly significant in tissues like the brain, which have limited energy reserves and weaker antioxidant defenses. For instance, neurons have much lower levels of a key antioxidant catalase compared to hepatocytes [11].

The brain meets its high demands for maintaining redox homeostasis through two distinct mechanisms in neurons and astrocytes, which communicate with each other. Astrocytes are crucial in providing antioxidant support to nearby neurons, facilitated by a broadly expressed nuclear erythroid 2-related factor 2 (Nrf2) that regulates gene expression of various antioxidant enzymes. However, recent data suggest that while most cells express genes for Nrf2 with activity determined by ROS levels, neurons actively avoid Nrf2 activity by keeping its gene expression low [13,14]. Instead, neurons rely on the activity-dependent control of numerous antioxidant genes. This allows them to counteract ROS formation by boosting the antioxidant capacity of both the glutathione and thioredoxin/peroxiredoxin systems [15,16].

Ischemia-reperfusion (IR) injury is a common feature of strokes, characterized by an initial period of reduced blood flow followed by its restoration. The brain is highly susceptible to ischemia; it ceases to function within seconds, and necrotic processes begin after 5 min of complete deprivation of oxygen and glucose [17,18]. Current studies have focused on understanding the molecular and cellular mechanisms that protect the brain from subsequent ischemic injury. One potent protective mechanism is ischemic preconditioning (IPC). Previous investigations have shown that IPC can significantly enhance neuronal survival following temporary critical ischemia [19]. Although the molecular mechanisms are still not fully understood, recent studies have identified several key molecules and proteins crucial for the development of ischemic tolerance in the brain. Fundamental signaling pathways involved include the activation of N-methyl-D-aspartate receptor, mitogen-activated protein kinases, heat-shock proteins, low-level ROS generation, peroxiredoxin-related pathways, ubiquitin–proteasome pathways, and autophagy–lysosomal pathways [20,21].

This theoretical knowledge opens possibilities for utilizing the potential benefits of IPC for the prevention and treatment of brain damage due to ischemia. Hence, the current study investigates the effects of cerebral IR and IPC on energy status and redox-active molecules, specifically focusing on the peroxiredoxin and glutathione systems within the cortex and hippocampus.

## 2. Materials and Methods

### 2.1. Animals

White male Wistar rats (Velaz, Prague, Czech Republic) at the age of 3–4 months with a mean weight of 335.0 ± 42.7 g were randomly divided into three groups: control (CON, *n* = 11), ischemia–reperfusion (IR, *n* = 11), and after ischemic preconditioning (IPC, *n* = 11). Animals were kept in a temperature-controlled room at 22 ± 2 °C with a 12 h light/dark cycle, with access to food and water ad libitum. All experiments were performed following the EU directive (2010/63/EU) concerning the protection of animals used for scientific purposes and approved by the Ethical Committee of Comenius University in Bratislava, Jessenius Faculty, in Martin (No. 1647/2015, 9. August 2016) and by the State Veterinary and Food Department of the Slovak Republic (No. 2857/16-221).

### 2.2. Induction of Cerebral Ischemia-Reperfusion and Ischemic Preconditioning

Animals were placed in an anesthetic box and initially anesthetized with 4.5% sevoflurane in a mixture of 33% O_2_ and 66% N_2_O, while during the surgery the concentration of sevoflurane was maintained around 3–3.5%. The body temperature of the animals was maintained during the procedures using a thermostatically heated pad. During the reoxygenation period, animals were monitored at regular intervals. Rats in the IR group underwent 15 min of global forebrain ischemia induced by a standard 4-vessel occlusion model developed by Pulsinelli and Brierley [22] and were subsequently decapitated after a 24 h reperfusion period. In the IPC group, animals were subjected to 5 min global forebrain ischemia 48 h before the ischemic intervention described above. The control group underwent a sham ischemia operation, meaning that vessels were not occluded. Subsequently, the brain (*n* = 6 per group) was removed and divided into the cortex and hippocampus. The remaining 5 brains were dissected and processed immediately for immunohistochemical analysis, which is explained in Section 2.9.

### 2.3. Tissue Homogenisation, Isolation of Mitochondria, and Protein Content Determination

Tissue homogenates were prepared from the cerebral hippocampus and cortex separately. Frozen tissue was thawed in 10 volumes of ice-cold homogenization buffer (5 mM HEPES pH 7.0, 0.32 M sucrose, 1 mM EDTA, 0.3 mM phenylmethylsulfonyl fluoride) and homogenized (1300 rpm/min, 5× in 30/20 s intervals) using precooled Teflon-glass Potter-Elvehjem homogenizer (Sartorius, Goettingen, Germany). Homogenates were stored as 100 µL aliquots. A mitochondrial fraction from cortex homogenates was isolated by differential centrifugation according to the original method [23]. Firstly, the homogenate was centrifuged at 1000× *g* for 10 min at 4 °C. The supernatant was fractionated by sucrose density gradient centrifugation at 100,000× *g* for 20 min. The resulting mitochondrial fraction was resuspended in 0.5 mL of 0.32 M sucrose and stored at −80 °C for further experiments.

Protein concentration in each sample was determined using the commercially available DC Protein assay kit (500-0111; Bio-Rad Laboratories, Hercules, CA, USA). The measurement was performed according to the manufacturer’s instructions using a BioTek Synergy H4 hybrid microplate reader (Agilent Technologies, Santa Clara, CA, USA) and bovine serum albumin (BSA) as a standard.

### 2.4. Western Blot and Immunodetection

Proteins (30 µg per sample) from tissue homogenates/mitochondria (*n* = 4 per subgroup) were separated using sodium dodecyl sulfate-polyacrylamide gel electrophoresis (SDS-PAGE) with 8%, 12%, or 15% gels (based on Mw of detected protein) under denaturing conditions (3 min at 95 °C) and then transferred (a semi-dry transfer) to nitrocellulose membranes (0.45 μm) using Trans-Blot^®^ SD Semi-Dry Transfer Cell apparatus (1620115; Bio-Rad Laboratories, Hercules, CA, USA) at 60 mA per gel for 80 min. To quantify the total protein, the resulting blots were incubated in red-colored Ponceau S for 5 min. Non-specific binding was blocked with 2% BSA in TBS-T (Tris’s base saline and 0.05% Tween 20) during the night.

Next day, the membranes were washed 3× 7 min in TBS-T (the solution was used between each step) and incubated at 4 °C overnight in an orbital-rocking shaker Polymax 2040 (Heidolph Instruments, Schwabach, Germany) with the following HRP-conjugated primary antibodies purchased from Santa Cruz Biotechnology (Dallas, TX, USA): anti-ATPB (sc-55597, 1:1000), anti-HSP60 (sc-59567, 1:500), anti-PRX5 (sc-133073, 1:100), anti-PRX6 (sc-393024, 1:100), anti-SOD2 (sc-133134, 1:1000). Anti-thioredoxin 2 (14907, 1:1000) and β-actin (3700S, 1:10,000) were obtained from Cell Signaling Technology (Danvers, MA, USA), while anti-PRX-SO3 (ab16830, 1:1000), and anti-Total OXPHOS Cocktail (ab110413, 1:1000) were from Abcam (Cambridge, UK). Then, membranes were incubated with the appropriate secondary antibodies for 1 h: goat anti-mouse IgG-HRP (sc-2005, 1:2000; Santa Cruz Biotechnology, Dallas, TX, USA) was used for ATPB, Beta-actin, HSP60, PRX5, PRX6, SOD2, TRX2, and Total OXPHOS Cocktail; goat anti-rabbit IgG-HRP (A9169, 1:20,000; Sigma-Aldrich, Munich, Germany) for PRX-SO3.

Finally, the membranes were washed with TBS-T and then incubated in SuperSignal West Pico PLUS Chemiluminescent Substrate (ThermoFisher Scientific, Waltham, MA, USA) solution for 5 min in the dark. After the exposition, corresponding bands were visualized using a ChemiDoc^™^ XRS Imaging System and quantified in the Image Lab^™^ Software 6.0.1 (both Bio-Rad Laboratories, Hercules, CA, USA). The obtained results were normalized to a loading control beta-actin for homogenate and HSP60 for mitochondria. The results are expressed as relative protein levels in arbitrary units (AU).

### 2.5. Two-Dimensional Electrophoresis (2-DE)

From each cortex mitochondria and hippocampus sample (*n* = 6/subgroup), 250 μg of protein content was precipitated in ice-cold acetone, resuspended in 0.2 mL of Rehydration/Sample buffer (163-2106, Bio-Rad Laboratories, Hercules, CA, USA), and applied to Ready Strip IPG gel (11 cm, linear gradient between pH 3–10; Bio-Rad Laboratories, Hercules, CA, USA). The first dimension of the immobilized pH gradient of the IPG gel was carried out according to the manufacturer’s instructions.

After the first dimension, the IPG gel was equilibrated with Equilibration Buffer I (163-2107; Bio-Rad Laboratories, Hercules, CA, USA) for 15 min and further equilibrated an additional 15 min with Equilibrium Buffer II (163-2108; Bio-Rad Laboratories, Hercules, CA, USA) and 0.03 g/mL of iodoacetamide. Then, the IPG gel was subjected to second-dimensional SDS-PAGE separation using 12% gel. All the gels were stained with Bio-Safe Coomassie blue G-250 and images were captured by scanning the gels using GS-800 Calibrated Densitometer and densitometrically analyzed using PDQuest 8 software (all Bio-Rad Laboratories, Hercules, CA, USA).

### 2.6. Protein Identification by Mass Spectrometry

Protein spots were excised from 2-DE gels, washed with 50% acetonitrile (ACN) until the blue color was removed, and dehydrated with 100% ACN. Then, the spots were incubated for 45 min at 56 °C with 10 mM dithiothreitol solution (disulfide bridge reduction) followed by incubation in 55 mM iodoacetamide for 30 min in the dark to alkylate cysteine residues, and finally washed 2× with 25 mM ammonium bicarbonate (ABC) and 100% ACN alternatively. For in-gel digestion, 3 µL of trypsin solution (20 ng/µL in ABC) was added, incubated for 30 min on ice, and digested at 37 °C overnight. Peptides were extracted with 10% trifluoroacetic acid, spots were dehydrated (100% ACN), and collected samples were concentrated under a vacuum (Concentrator plus/Vacufuge plus; Eppendorf, Leipzig, Germany) until a final volume of 10 µL was obtained. When the spotted sample (0.75 µL) aliquot was dried down on the MALDI (matrix-assisted laser desorption ionization) target (AnchorChip Standard; Bruker Daltonics, Bremen, Germany), the same amount of matrix (α-cyano-4-hydroxycinnamic acid, 1 mg/mL) solution was added and left to dry down.

Mass spectra and MS/MS fragmentation spectra were acquired using the MALDI TOF UltrafleXtreme (Bruker Daltonics, Bremen, Germany) in the 700–3500 kDa m/z range. For MASCOT (Matrix Science) search, we used the following parameters: taxonomy—*Rattus norvegicus*, mass tolerance for PMF—50 ppm and one missing cleavage, MS/MS tolerance—0.5 Da, global modification—carbamidomethyl (C), and variable modification—methionine oxidation (M). The masses of the observed peptides using MASCOT were queried against the SwissProt and NCBIprot databases to determine and confirm the identity.

### 2.7. Quantification, Protein–Protein Interaction Network

The differentially expressed protein cutoff value was set above a 1.5-fold increase for upregulated proteins and a 0.67-fold decrease for downregulated proteins. Statistical significance (*p* < 0.05) was determined using Student’s *t*-test.

Protein–protein interaction network analysis was performed using the search tool for retrieval of interacting genes—STRING database https://string-db.org (accessed on 6 March 2024), which integrates known and predicted protein–protein interactions and can be applied to predict functional interactions of proteins [24].

### 2.8. Total Glutathione and the Assay of Enzyme Activities

#### 2.8.1. Glutathione Content, Glutathione Reductase, and Glutathione Peroxidase Activity

All parameters were measured in hippocampal homogenates using commercial kits purchased from Sigma-Aldrich (Munich, Germany)—Glutathione Assay Kit (S0260), Glutathione Reductase Assay Kit (GRSA), and Glutathione Peroxidase Assay Kit (CGP1) according to the manufacturer’s instructions. Changes in absorbance were measured using a BioTek Synergy H4 hybrid microplate reader (Agilent Technologies, Santa Clara, CA, USA).

#### 2.8.2. The Assay of Superoxide Dismutase and ATP Synthase Activity

Antioxidant superoxide dismutase activity (SOD) was determined in the cortical mitochondria as previously described by [25]. The activity of ATP synthase was determined according to the slightly modified method [26] using a coupled reaction of ADP production with the oxidation of NADH. The reaction was measured spectrophotometrically at 340 nm (ε = 6.2 M.cm^−1^). Hippocampal proteins (5 µg/mL) were added to the working medium (250 mM sucrose, 50 mM KCl, 20 mM Tris-HCl pH 7.4, 2 mM MgCl_2_, 2 mM phosphoenolpyruvate, 10 µg/mL lactate dehydrogenase, 20 µg/mL pyruvate kinase, 20 µM rotenone, and 0.2 mM NADH). The addition of 2.5 mM ATP initiated a reaction.

### 2.9. Fluorescent Immunohistochemistry

Animals from CON, IR, and IPC (*n* = 5 per subgroup) were placed in an anesthetic box and put to sleep as described above. Animals were subsequently trans-cranially perfused with 0.1 M phosphate-buffered saline buffer (PBS, pH 7.4) followed by 4% paraformaldehyde in 0.1 M PBS (pH 7.4). After perfusion, all animals were decapitated, and the brains were removed from the skull and submerged overnight in the same fixative at 4 °C, and placed in 30% sucrose for the next 24 h at 4 °C. The rat brains were embedded with a specific embedding medium (Killik, Bio Optica, Milano, Italy) and promptly frozen by a fast-cooling boost in Shannon Cryotome E (Thermo Scientific, Waltham, MA, USA), sectioned into 30 μm thick coronal slices, using the stereotaxic coordinates of the rat brain atlas of Paxinos and Watson [27] as a reference. The coordinates (from Bregma) were approximately −2.8 mm to −3.8 mm. Brain sections were permeabilized with 0.1% Triton X-100, and blocked for 1 h with 10% BSA. The tissue sections were incubated at 4 °C overnight using primary antibodies (PRX5, PRX6, and PRX-SO3), all diluted 1:100 in the 0.1% Triton X-100 enriched with 10% BSA. Immunofluorescent detection was accomplished using Alexa Fluor 488 goat anti-mouse IgG (A11001, 1:100; Thermo Scientific Waltham, MA, USA) secondary antibody. Sections were mounted with DAPI Fluoromount-G^®^ (0100-20; SouthernBiotech, Birmingham, AL, USA) according to the standard protocol. No immunoreactivity was detected in the absence of the primary antibody.

The slides were examined by a confocal laser scanning microscope, Olympus FluoView FV10i (Olympus, Tokyo, Japan) in the *cornu ammonis* 1 (CA1), CA3, and *gyrus dentatus* (GD) of the hippocampus and the primary motor cortex (M1) area of the rat brains. The objective of 10× with zoom up to 60× magnification equipped with filters for Alexa Fluor 488 (excitation: 499 nm; emission: 520 nm) was utilized. The image capture was performed with Olympus Fluoview FV10-ASW software, version 02.01 (Olympus, Tokyo, Japan), Quick Photo Micro software, version 2.3 (Promicra, Prague, Czech Republic), and further processed in Adobe Photoshop CS3 Extended, version 10.0 for Windows (Adobe Systems, San Jose, CA, USA).

### 2.10. Data Analysis

All statistical analyses were performed using GraphPad InStat V3.01 (GraphPad Software, Boston, MA, USA). Data are presented as mean ± standard error of the mean (SEM). For the comparisons of IR or IPC changes among all groups, a one-way ANOVA with post hoc comparisons by the Student–Neuman–Keuls test was carried out to test for differences between experimental groups. A value of *p* < 0.05 was considered as statistically significant.

## 3. Results

### 3.1. Identification of Altered Proteins in the Cortex and Hippocampus

**Figure 1:**
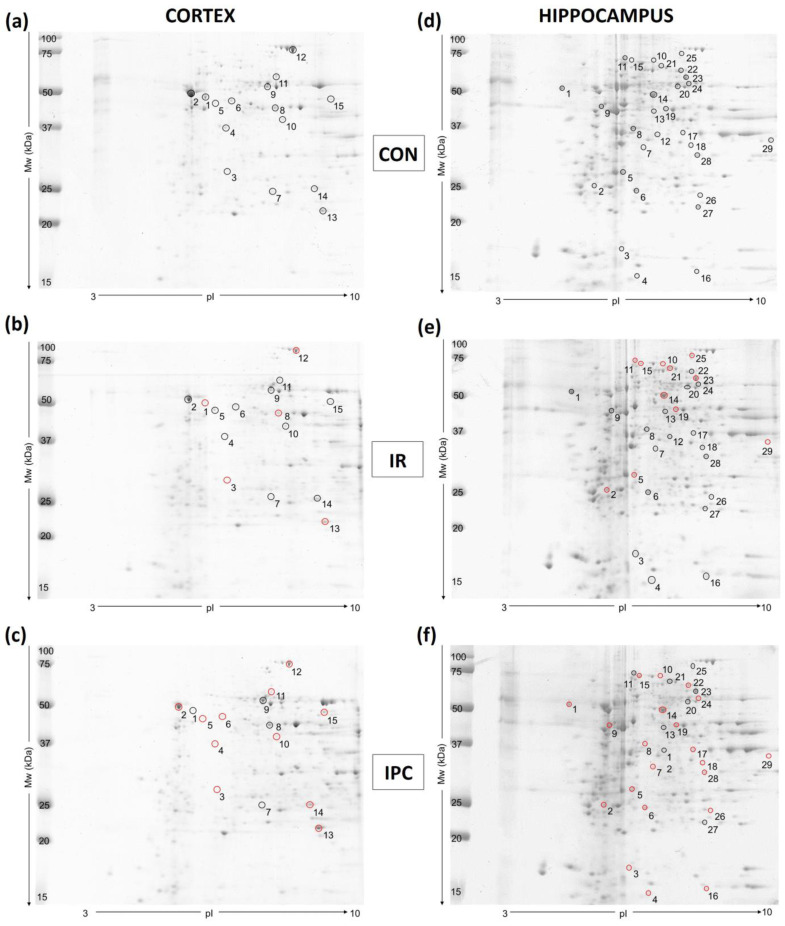
Representative 2-DE gels for (**a**) CON; (**b**) IR; (**c**) IPC groups in cortex mitochondria as well as for (**d**) CON; (**e**) IR; (**f**) IPC groups in the hippocampus. The cutoff value for differentially expressed proteins was set at a fold change above 1.5, with a 95% confidence interval, and *p* < 0.05 considered a statistically significant change in protein level. The gray circle (◦) represents all protein spots significantly modified as shown in the control 2-DE gels. Red circles (◦) represent the elevation of protein content compared to the control group in the respective tissue. CON (control), IR (ischemia-reperfusion), IPC (ischemic preconditioning).

The brain’s use of diverse reactive species for signal transmission makes it susceptible to oxidative stress, which is detrimental to normal brain functioning. Proteins are among the first targets for potential modification. Therefore, we have been looking for proteins with at least a 1.5-fold change in density (*p* < 0.05) and a 95% confidence interval between experimental groups of cortex mitochondria and the hippocampus (Figure 1). The 2-DE protein maps from both brain areas showed a different level of vulnerability to cerebral ischemia. From the initial pool of 2-DE identified proteins in cortex mitochondria (*n* = 251) and in the hippocampus (*n* = 304), only 15 individual proteins were modified in cortex mitochondria and 29 in the hippocampus after IR injury and IPC, respectively.

**Figure 2:**
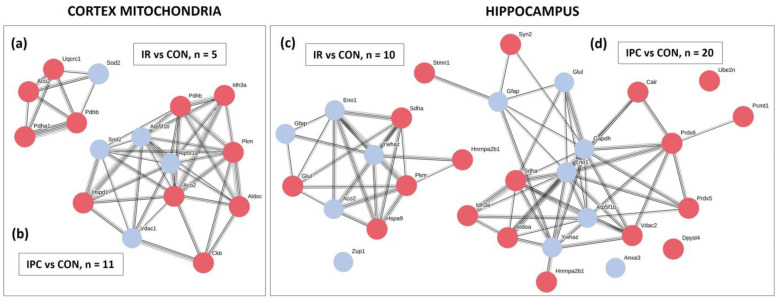
String protein–protein interaction maps of modified proteins in cortex mitochondria and hippocampus. Interaction map showing (**a**) changes in content of 5 modified proteins after IR in cortex mitochondria; (**b**) changes in content of 11 modified proteins after IPC in cortex mitochondria; (**c**) changes in content of 10 modified proteins after IR in the hippocampus; (**d**) changes in content of 20 modified proteins after IPC in the hippocampus. Line thickness between proteins indicates the strength of data support. Colors represent upregulated (•) or downregulated (•) proteins between experimental groups. CON (control), IR (ischemia-reperfusion), IPC (ischemic preconditioning).

More detailed information about each identified protein is summarized in Appendix A for cortex mitochondria (Table A1) and hippocampal proteins (Table A2). All the changes are also presented as string protein–protein interaction maps (Figure 2) that show protein connections and highlight their upregulation (blue dots) and/or downregulation (red dots). Interaction maps show in the cortex mitochondria 5 modified (1 upregulated) proteins after IR injury, and 11 modified (4 upregulated) proteins after IPC; in the hippocampus are shown 10 modified (5 upregulated) proteins after IR injury, and 20 modified (7 upregulated) proteins after IPC. Line thickness between proteins indicates the strength of data support.

In the cortex, mitochondria-specific superoxide dismutase (SOD2) was the only protein elevated after both conditions, with IPC inducing a significant (* *p* < 0.05), 2.26-fold increase in protein content. In contrast, downregulation of cytochrome bc1 complex subunit 1, aconitate hydratase, pyruvate dehydrogenase E1 subunit alpha, and pyruvate dehydrogenase E1 subunit beta was detected in mitochondria subjected to IR (Figure 2a). In addition to SOD2, ischemic preconditioning caused the upregulation of voltage-dependent anion channel 1, ATP synthase subunit alpha, and beta from the initially modified 11 proteins (Figure 2b).

In the hippocampus, the IPC-stimulated increase in glyceraldehyde-3P dehydrogenase and glutamine synthetase was accompanied by the downregulation of proteins related to the energy-producing machinery, such as isocitrate dehydrogenase 3 (NAD+) alpha, succinate dehydrogenase (ubiquinone) flavoprotein subunit, and glycolytic aldolase A. While cerebral IR did not affect the content of antioxidant molecules in the hippocampus, the neuron-specific peroxiredoxin 5 (PRX5) level dropped by 1.85-fold in the IPC group compared to the control. Another member of the peroxiredoxin family, PRX6 exhibited more pronounced changes, with a 2.78-fold decrease observed after induced IPC (Figure 2d; for details, see Table A2).

### 3.2. Western Blot Analysis and Immunodetection of Modified Proteins

**Figure 3:**
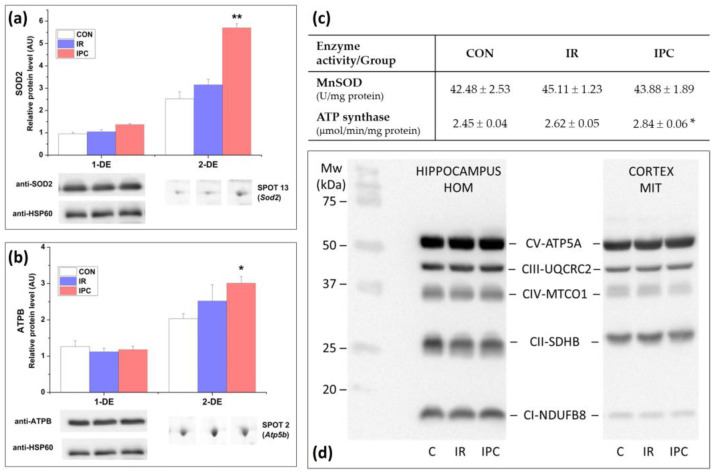
The effect of IR and IPC on the amount and activity of selected proteins. (**a**) Changes in SOD2 protein level after 1-DE and 2-DE with quantification in the cortex mitochondria; (**b**) changes in ATPB protein level after 1-DE and 2-DE with quantification in the cortex mitochondria; (**c**) changes in the activity of MnSOD and ATP synthase in cortex mitochondria; (**d**) representative Western blots for detection of oxidative phosphorylation complexes’ core subunit protein level in cortex mitochondria and hippocampus. Values are expressed as mean ± SEM of 4 samples per each experimental group (1-DE) or 6 samples per each experimental group (2-DE, enzyme activity assay). A one-way ANOVA with post hoc comparisons by the Student–Neuman–Keuls test was carried out to test for significant differences (*) when compared to the control: * *p* < 0.05, ** *p* < 0.01. ATPA (ATP synthase subunit alpha), ATPB (ATP synthase subunit beta), CON (control), IR (ischemia-reperfusion), IPC (ischemic preconditioning), HOM (homogenate), MIT (mitochondria), MnSOD/SOD2 (mitochondrial Mn superoxide dismutase), MTCO1 (mitochondrially encoded cytochrome c oxidase subunit I), NDUFB8 (NADH: ubiquinone oxidoreductase subunit B8), SDHB (succinate dehydrogenase subunit beta), UQCRC2 (ubiquinol–cytochrome c reductase core protein II).

Western blot analysis, combined with analysis of specific antibodies (1-DE), was conducted to monitor and verify changes observed in 2-DE. Although the SOD2 protein level in cortex mitochondria increased by 54.5% in the IPC group compared to the control, these changes were not statistically significant after normalization. This is supported by maintained SOD activity in mitochondria (Figure 3a,c). Another 2-DE upregulated protein spot, ATPB, was not affected by IR or IPC (Figure 3b). Given that 2-DE revealed the cortex mitochondria’s heightened sensitivity to IPC, characterized by the deprivation of NADH-producing dehydrogenases, it may impact mitochondrial respiration and ATP production. We detected maintained protein content not only for the alpha subunit of ATP synthase, whose activity was enhanced by 16.6% (Figure 3c), but also for other core subunits of respiratory chain complexes in the cortex mitochondria and hippocampus after IR and IPC (Figure 3d).

**Figure 4:**
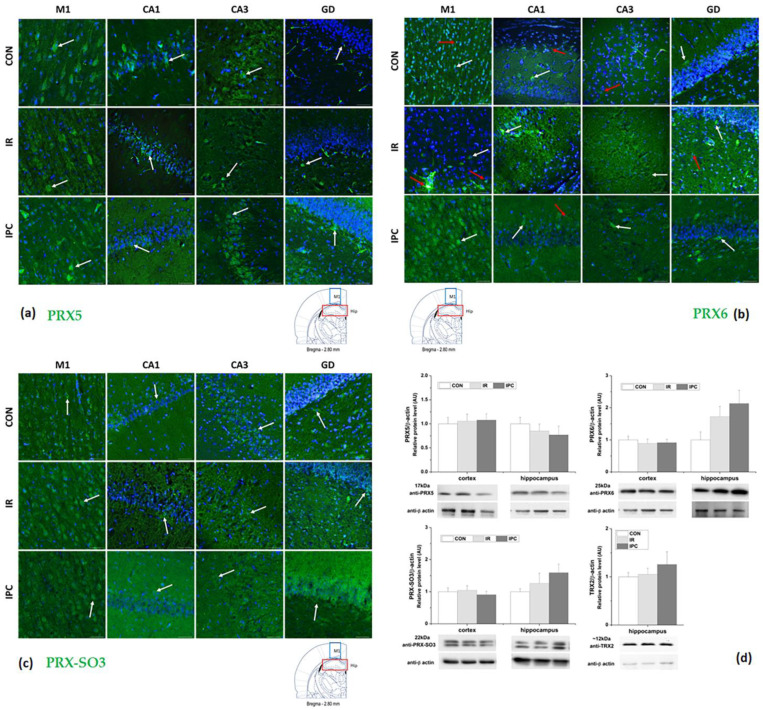
Representative fluorescence micrographs show (**a**) peroxiredoxin 5 (PRX5); (**b**) peroxiredoxin 6 (PRX6); (**c**) sulfonylated peroxiredoxin peptide (PRX-SO3) positivity in the *cornu ammonis* 1 (CA1), CA3, and *gyrus dentatus* (GD) of the hippocampus and the primary motor cortex (M1) area of rat brains (5 samples per each group) subjected to the IR and IPC. White arrows indicate positive cells and red arrows are PRX6 positive astrocytes. Nuclei were labeled with blue DAPI. Scale bar = 50 μm; (**d**) Western blots for PRX5, PRX6, PRX-SO3, and thioredoxin 2 (TRX2) detected in IR and IPC groups. Results in the bar graphs are presented as the relative protein content normalized to β-actin and compared to the basal control value. Graphs show the mean ± SEM for at least 3 replicates. A one-way ANOVA with post hoc comparisons by the Student–Neuman–Keuls test was carried out to test for significant differences when compared to the control. CON (control), IR (ischemia-reperfusion), and IPC (ischemic preconditioning).

When samples were subjected to immunoblot analysis with antibodies to the sulfonylated peroxiredoxin peptide (PRX-SO3), the elevation in protein content was detected after IPC in the hippocampus, while the cortex lysate showed a decrease in quantity. No significant changes were observed in IR groups from both the cortex and the hippocampus. In neurons, the widely expressed PRDX5 isoform did not change after IR and IPC in both tissues. The PRDX6 isoform was the last protein detected as modified in the 2-DE, showing 2.1-fold upregulation (* *p* < 0.05) after immunoblot detection in the IPC group compared to the control in the hippocampus (Figure 4d).

Peroxiredoxins, as a family of peroxidases, catalyze the reduction of hydrogen peroxide and/or alkyl hydroperoxides. This process utilizes reducing equivalents provided by thiol-containing proteins, such as thioredoxin. In our experiments, immunoblot analysis of mitochondria-specific thioredoxin 2 (TRX2) revealed stable protein levels in hippocampal cells of both the IR and IPC groups compared to controls. It seems that cerebral ischemia sensitizes hippocampal cells which, upon exposure to additional short ischemia, accelerate changes.

### 3.3. Fluorescent Immunohistochemical Analysis of Peroxiredoxins

The family of peroxiredoxins represents thiol-specific peroxidases with the ability to detoxify reactive oxygen species. Peroxiredoxins modulate various receptor signaling pathways and protect cells from oxidatively induced death. While 2-DE analysis revealed higher sensitivity to ischemic injury of PRX5 and PRX6, these changes were not confirmed by immunoblotting analysis. However, peroxiredoxins have a certain level of specificity and distribution. Our model of global cerebral IR demonstrates differentially expressed and localized PRX5, PRX6, and hyperoxidized PRX-SO3 in three areas of the hippocampus (CA1, CA3, and GD) and the primary motor cortex (M1) area of rat brains (Figure 4).

In the histologically intact tissue (control group, CON), PRX5 was predominantly located within the cytoplasm of perikarya and processes of neurons, mainly axons (Figure 4a). When comparing intensities of fluorescent signals from the cortex and hippocampus, the highest immunoreactivity was detected in the M1 region of the cortex. In the IR group, we detected a decrease in fluorescent signal in CA1 of the hippocampus, with sight decrease in hippocampal CA3. In the GD area, we found a fluorescent signal in different neuronal types as in controls. It seems that positive cells were mostly found in the extra-granular layers, likely representing interneurons or intermediate progenitor cells in the hilus. Ischemic preconditioning resulted in increased PRX5 fluorescence in the CA3 and GD regions of the analyzed hippocampal areas. The most intensive staining was observed in the GD, indicating a higher number of positive interneurons or intermediate progenitor cells in the extra-granular layer of the hilus. Additionally, an increase in fluorescence intensity was detected in the M1 cortical area.

The highest immunoreactivity to PRX6 was detected also in the M1 region of the cortex (Figure 4b) and the GD region in the hippocampus. In the IR group, we observed a decrease in fluorescent signal in the CA3 region, with a slight increase in CA1. Within the GD area, we observed a fluorescent signal in various neuronal types. Positive cells were observed in both the granular and extra-granular layers, suggesting the presence of interneurons. Our results demonstrate a decrease in PRX6 positivity in astrocytes (indicated by red arrows) and an increase in positive neurons following IR. A similar pattern was observed in the group exposed to IPC, wherein the highest overall PRX6 fluorescence was detected in the M1 cortical area. Hippocampal areas exhibited selectively higher positivity, with the most intensive staining observed in the GD region.

In histologically intact tissue, hyperoxidized PRX-SO3 was uniformly distributed within the plasmalemma of perikarya. Upon comparing fluorescent signal intensities, we detected the highest immunoreactivity in the M1 region of the cortex. In the hippocampus, the GD region exhibited the highest intensity of the fluorescent signal. In the IR group, we observed a decrease in the CA1 and CA3 regions of the hippocampus, accompanied by a slight increase in the GD area, like the distribution observed in the control group. It seems that positive cells were in their extra-granular layers, but we found a slightly increased positivity in the granular layer. IPC caused a decrease in PRX-SO3 fluorescence in the M1 cortical area. Conversely, there was a significant increase in fluorescence observed across all hippocampal areas, with the most intense staining noted in the GD region. Furthermore, there was a higher number of positive granular cells and structures in the molecular layer.

The activity of important antioxidant molecules was measured to further investigate changes and their possible impact on peroxiredoxins in the hippocampus. Interestingly, no significant changes were detected in the activity of superoxide dismutase (Table 1) or glutathione peroxidase activity after both IR and IPC in the hippocampus.

The total glutathione content significantly dropped by 16.82% from 106.57 ± 8.57 to 88.65 ± 20.72 nmol/mg protein in the group subjected to IPC when compared to the controls. In contrast, the activity of glutathione reductase, an enzyme responsible for the regeneration of the oxidized glutathione, was stimulated significantly from the initial activity from 3.83 ± 1.99 to 14.05 ± 1.61 μmol/min/mg protein in the IPC group, while IR did not affect glutathione reductase activity in hippocampal cells.

## 4. Discussion

Brain ischemia, resulting either from global or focal decreases in perfusion, contributes significantly to disability and mortality in a wide range of pathologies, including cardiac arrest, trauma, stroke, and acute brain ischemia-reperfusion (IR) injury is a major pathophysiological manifestation [28]. Studies have shown that the body’s own molecular and cellular mechanisms can provide some degree of protection from brain ischemia [15,29,30]. Ischemic preconditioning (IPC), brief episodes of nonlethal ischemia before the ischemic injury, offers a way to induce endogenous neuroprotection [31] to prevent or at least significantly reduce ischemic brain damage. Now, it is regarded as one potential therapeutic strategy, but the basic molecular biology mechanisms of IPC are still unclear [32] despite years of study of the brain [33,34,35,36] and heart [37,38,39].

Since the brain has abundant lipid content, high energy requirements, and weak antioxidant capacity, it is an easy target for excessive oxidative insults [40]. Therefore, the brain even in basal conditions requires effective strategies to cope with oxidative stress, which ultimately leads to the modification of biomolecules such as proteins, which carry out the most essential cellular functions [40,41,42]. The proteome is highly dynamic and adapts in response to changes in the environment; therefore, its composition bears critical information related to the actual state of the organism. The 2-DE protein maps and protein-protein interactions showed a different level of vulnerability to cerebral IR concerning the different brain areas.

Cortex mitochondria play a crucial role in maintaining an adequate energy supply essential for brain functioning. In our study, the significant upregulation of the main mitochondria-specific enzyme, manganese superoxide dismutase (SOD2), during IR and even more prominently by 2.26-fold following IPC, aided in mitigating the excessive flow of ROS during reperfusion. SOD2 primarily catalyzes the dismutation of superoxide radicals to H_2_O_2_, which is relatively stable under physiological pH, temperature, and in the absence of metal ions [43,44]. In addition to SOD2, IPC caused the upregulation of voltage-dependent anion channel 1, ATP synthase subunit alpha, and beta to help stabilize overall energy machinery. These findings overlap with a study focused on protein profiling of acute ischemic stroke in rats [45]. Immunoblots confirmed the sustained levels of SOD2 and enzymatic activity in cortical mitochondria following both IR and IPC. These results are consistent with a study in which IPC did not induce changes in SOD expression and activity [46].

Consistent results were observed in a study where the maintenance of SOD2 induced by IPC was compromised by the depletion and/or absence of TRX2. Furthermore, this study demonstrated that IPC conferred neuroprotection against ischemic injury by preserving TRX2 levels, especially in the hippocampal CA1 region [47]. In our experiments, immunoblot analysis of TRX2 revealed stable protein levels. This is important, since SOD2 knockout mice die shortly after birth due to increased oxidative stress, while mice lacking SOD1 have a normal lifespan [48]. Moreover, based on SOD2 location, modification of this protein can lead to greatly impaired proteasome function [49,50].

Healthy regulation of mitochondrial redox and energy metabolism along with proteolytic pathways are found to be critical in ROS handling and promotion of neurodegeneration [51]. The ATP synthase subunit beta, identified through 2-DE, is another upregulated protein following IPC. This protein subunit serves as a site for ATP synthesis. The activity of ATP synthase in cortex mitochondria was significantly enhanced by 16.6% following IPC. Interestingly, core subunits of respiratory chain complexes in both tissues remained stable after IR and IPC. It has been described that depending on the oxidative/nitrosative modifications, ATP synthase can dynamically modulate its activity, transitioning between catalytic and pore-forming states [52].

Hippocampal proteins in comparison to the cortex were highly sensitive to ROS attack with a negative impact on defense mechanisms leading to dysregulation of energy, metabolism mainly in the IPC group, when compared to the control. The 2-DE analysis revealed higher sensitivity of peroxiredoxin 5 (PRX5) and PRX6 to IPC. Immunoblot analysis showed no changes in PRX5 content following both IR and IPC in both tissues. PRX6 content was found to be upregulated by 2.1-fold after IPC specifically in the hippocampus. Peroxiredoxins, functioning as a family of peroxidases, play a crucial role in catalyzing the reduction of H_2_O_2_ and/or alkyl hydroperoxides [53]. While the atypical 2-Cys PRX5 forms intramolecular disulfide bonds and requires the oxidoreductase activity of TRX for reduction, the 1-Cys PRX6 utilizes glutathione, ascorbate, and lipoic acid as reductants, but not TRX [54]. Moreover, overoxidation of PRXs leads to the formation of high-molecular-weight complexes, which acquire molecular chaperone activity, thus safeguarding cells against stress-induced protein unfolding [55]. In addition to their peroxidase and chaperone activities, PRXs interact with other proteins, thereby participating in various intracellular processes such as neuronal differentiation, cell growth, or apoptosis [56]. This PRX–protein interaction might direct PRX cellular localization and sensitivity to oxidation, especially in situations with high local H_2_O_2_ accumulation. One of the mechanisms comprises the regulation of PRX via phosphorylation, which enables its function as an H_2_O_2_ sensor if the oxidation state of the PRX cysteine active site can be transferred to other proteins that are less susceptible to H_2_O_2_ [57].

Peroxiredoxins exhibit specific distribution patterns. PRX1, 2, and 6 are mainly located in the cytosol; PRX3 is restricted to the mitochondria, while PRX5 has been identified in both neuronal mitochondria and cytosol. PRX4 is in the endoplasmic reticulum and secreted into the extracellular environment [58,59]. In the histologically intact tissue, PRX5 was predominantly located within the cytoplasm of perikarya and processes of neurons, mainly axons. The highest immunoreactivity was detected in the M1 region of the cortex. Previous reports indicate that pyramidal cells in the CA3 region and granule cells in the GD region exhibit relative resistance to ischemia [60], while the CA1 region is more vulnerable [61], especially after 10 min of hippocampal ischemia triggering selective pyramidal neuronal loss in rats [60]. Low basal expression of PRXs along with the various modifications detected was suggested to contribute to the higher vulnerability of hippocampal neurons in neurodegenerative disorders [62]. Increased ROS can cause neuronal cell death in the CA1 of the hippocampus and its functional changes, such as learning and memory impairment [63]. Notably, oxidative and nitrosative changes, along with phosphorylation of PRXs, have been detected in the *substantia nigra* of Parkinson’s disease in both animal models and human subjects [62,64], resulting in deactivation of PRXs. It was found that PRX2 deletion impairs hippocampal-dependent memory via aggravation of cerebral ischemia-induced oxidative damage in mice [63], while transgenic overexpression of PRX2 attenuated ischemic neuronal injury [65]. Injecting adenovirus-carrying PRX2 into the striatum a week before MPTP neurotoxic treatment notably prevented the loss of dopaminergic neurons in animals with Parkinson’s disease [64]. In our study, we observed the highest sensitivity to IR in the CA1 area. In the GD region, a positive fluorescent signal was detected in various neuronal types. Notably, these positive cells were mainly situated in the extra-granular layers, likely representing interneurons or intermediate progenitor cells in the hilus. The CA1 region exhibited the greatest vulnerability to preconditioning ischemia lasting 5 min. In contrast, IPC led to an overall increase in PRX5 fluorescence in the CA3 region and particularly intense staining in the GD region of the hippocampus, as well as in the M1 cortical area. The overexpression of PRX5 has been shown to mitigate the production of pro-inflammatory mediators TNF-α, IL-1β, and IL-6 [66], demonstrating its neuroprotective and anti-inflammatory properties. Maintaining the balance between degenerative processes, inflammatory surveillance, and neurogenesis may play a pivotal role in determining the long-term outcome of rats subjected to global ischemia [67]. In specifically sensitive brain areas, such as the hippocampal CA1, CA3, and M1 of the cortex, microglia and astrocytes exhibited significant activation. Conversely, in resistant brain areas like GD, only astrocytes were activated, resulting in less intensive neuroinflammation. Importantly, these neuroinflammatory effects persist for up to 2 years after brain IR injury, supported by continued microglia and astrocyte activity [67,68].

PRX6 has been detected in human and mouse brain astrocytes and at low levels in neurons but not in microglia [58,69]. Expression of PRDX6 has been observed in the oligodendrocytes of mice [58], although not in the human brain [69], but has been detected in the cerebrospinal fluid [70]. Using immunoblot analysis, an increase in the total PRX6 protein level was detected after IPC in the hippocampus. Fluorescent micrographs showed a decrease in PRX6 positivity in astrocytes accompanied by an increase in neurons following IR. A similar trend was observed in the group subjected to IPC, with the highest overall PRX6 fluorescence detected in the M1 cortical area and hippocampal GD region. Recently, it was found that extracellular PRX6 released by astrocytes triggers post-ischemic neuroapoptosis [71] and might induce protective astrogliosis in the ischemic brain [72]. PRX6 is a unique nonselenium glutathione peroxidase and acts as an acidic Ca^2+^-independent phospholipase A2 in astrocytes [73]. It plays a crucial role in repairing peroxidized cell membranes at physiological pH, a process augmented by reduced glutathione [74]. This function aids in the release of ischemia-activated PRX6 from damaged cells undergoing death, observed in both rats [75] and humans [76], and contributes to the protective function of astrocytes in amyloid β proteostasis [77]. Furthermore, in response to the loss of membrane potential, mitochondria recruit PRX6 to stabilize Pink1, a critical factor in the initial step of mitochondrial clearance known as mitophagy [78]. PRX6’s ability to reduce elevated intracellular Ca^2+^ level also suppresses cell death in astrocytes [74].

Aside from PRX5 and PRX6, 2-Cys PRX 1–4 has a rapid response to oxidative stress, forming hyperoxidized peroxiredoxins (PRX-SO3). In histologically intact tissue, PRX-SO3 was uniformly distributed within the plasmalemma of perikarya. In the IR group, we observed a decrease in the CA1 and CA3 regions, with a slight increase in the GD. Positive cells were mostly in their extra-granular layers. IPC resulted in lower PRX-SO3 fluorescence in the M1 cortical area, which was confirmed by immunoblot analysis; the PRX-SO3 protein content decreased in cortex lysates. Conversely, a significant increase in PRX-SO3 fluorescence was observed across all hippocampal areas, with the most intense staining in the GD region. Additionally, a higher number of positive granular cells and structures were observed in the molecular layer. With the same change, elevation of PRX-SO3 content was detected after IPC in the hippocampus. One study demonstrated a significant increase in PRX-SO3 immunoreactivity in the GD but not in CA1 or CA3 regions of the aging hippocampus [79]. Another study presented an elevation of PRX-SO3 positivity, which was detected 12 h and 24 h after cerebral IR [80].

Redox signaling before a lethal ischemic insult is an important step in triggering the protected state in IPC. The redox-active molecules were measured to further investigate changes and their possible impact on PRXs in the hippocampus. The activity of SOD and glutathione peroxidase (GPx) was maintained after both IR and IPC in the hippocampus. The same results where IPC did not induce any changes in SOD and GPx activities or their neuronal expression are shown in [46]. To cope with oxidative stress, the critical aspect is the ability of cells to synthesize glutathione and keep it reduced via glutathione reductase (GR) activity. We have observed a massive increase in GR activity in the hippocampus of animals exposed to IPC. This might be an adaptive mechanism in response to overloaded ROS.

## 5. Conclusions

Previous reports have suggested that several biomechanisms may be associated with the pathology of cerebral ischemic damage, such as loss of energy, ion imbalance, increased formation of ROS or apoptosis; events that may induce irreversible brain damage. Our study investigated the role of redox-active proteins in maintaining the energy supply. Protein profiling pointed out modified proteins related to energy and redox homeostasis. IPC preserved the integrity of cortex mitochondria, seen as enhanced SOD activity. Moreover, stable core subunits within respiratory chain complexes ensured adequate energy generation, supported by a rise in ATP synthase activity. In hippocampal cells, IPC increased PRX6 protein content and significantly enhanced GR activity to provide sufficient glutathione, thus stabilizing PRX6 function. The ability of astrocytes to mobilize PRX6 allows them to protect energy-producing neurons during initial ischemic events, as evidenced by a decrease in PRX6 positivity in astrocytes, accompanied by an increase in neurons following both IR injury and IPC. Maintained redox signaling via astrocyte–neuron communication may initiate a protective IPC state, and cooperation between PRX6, SOD, and glutathione reductase appears essential for safeguarding and stabilizing the hippocampus.

## Figures and Tables

**Table 1:** The activity of redox-active antioxidant molecules after IR and IPC in the hippocampus.

Antioxidant/Group	CON	IR	IPC
**SOD** (U/mg protein)	65.59 ± 0.94	66.12 ± 0.40	66.52 ± 1.07
**GR** (μmol/min/mg protein)	3.83 ± 1.99	5.51 ± 1.18	14.05 ± 1.61 ***
**GPx** (μmol/min/mg protein)	0.15 ± 0.03	0.19 ± 0.03	0.18 ± 0.02
**Total glutathione** (nmol/mg protein)	106.57 ± 8.57	103.19 ± 46.04	88.65 ± 20.72 *

Values are expressed as mean ± SEM of 6 samples per experimental group. A one-way ANOVA with post hoc comparisons by the Student–Neuman–Keuls test was carried out to test for significant differences (*) when compared to the control: * *p* < 0.05, *** *p* < 0.001. CON (control), IR (ischemia-reperfusion), IPC (ischemic preconditioning), SOD (superoxide dismutase), GR (glutathione reductase), GPx (glutathione peroxidase).

## Data Availability

All data related to this work can be made available upon request to the corresponding author.

## Appendix A

The appendix tables provide readers with all the details of data obtained from proteomic analysis. Each protein spot has a number that is linked with Figure 1 (2-DE protein maps of modified protein spots with at least a 1.5-fold change in density. Using mass spectrometry, proteins were identified using the ultrafleXtreme MALDI-TOF mass spectrometer, and the acquired protein ID, name, gene, Mw, and pI are summarized in Table A1 for proteins modified in the cortex mitochondria.

**Table A1:** Summary of mass spectrometry identification and quantitative analysis of modified proteins in cortex mitochondria subjected to IR injury and IPC.

Spot No.	Protein ID	Protein Name	Gene	Mw (kDa)	pI	IR/CON Fold Change/*p*-Value	IPC/CON Fold Change/*p*-Value
1	QCR1	Cytochrome b-c1 complex subunit 1, mitochondrial	*Uqcrc1*	53.50	5.57	0.34 0.011 *	ns
2	ATPB	ATP synthase subunit beta, mitochondrial	*Atp5b*	56.32	5.19	ns	1.54 0.033 *
3	ODPB	Pyruvate dehydrogenase E1 component subunit beta	*Pdhb*	39.30	6.20	0.49 0.0045 **	0.31 3 × 10^−4^ ***
4	IDH3A	Isocitrate dehydrogenase 3 (NAD+) alpha, mitochondrial	*Idh3a*	40.04	6.47	ns	0.53 0.053
5	KCRB	Creatine kinase, brain isoform CRA_a	*Ckb*	36.15	6.27	ns	0.35 0.043 *
6	CH60	60kDa heat shock protein, mitochondrial	*Hspd1*	61.09	5.91	ns	0.36 6 × 10^−4^ ***
7	TPIS	Triosephosphate isomerase	*Tpi1*	27.34	6.89	ns	ns
8	ODPA	Pyruvate dehydrogenase E1 subunit alpha, mitochondrial	*Pdha1*	43.88	6.67	0.57 0.012 *	ns
9	DLDH	Dihydrolipoyl dehydrogenase, mitochondrial	*Dld*	54.57	7.96	ns	ns
10	ALDOC	Fructose-bisphosphate aldolase C	*Aldoc*	43.88	8.49	ns	0.39 0.016 *
11	KPYM	Pyruvate kinase PKM	*Pkm*	58.29	6.63	ns	0.48 0.050
12	ACON	Aconitate hydratase, mitochondrial	*Aco2*	86.12	7.87	0.35 0.0014 **	0.27 1 × 10^−5^ ***
13	SODM	Superoxide dismutase [Mn], mitochondrial	*Sod2*	24.89	8.96	1.53 0.018 *	2.26 0.0086 **
14	ATPA	ATP synthase subunit alpha, mitochondrial	*Atp5a1*	59.83	9.22	ns	1.64 0.043 *
15	VDAC1	Voltage-dependent anion-selective channel protein 1	*Vdac1*	30.85	8.62	ns	2.85 0.018 *

* *p* < 0.05, ** *p* < 0.01, *** *p* < 0.001; significantly different when compared to the control.

Table A2 contains general information (all parameters) as well as results from statistical analysis of hippocampal proteins.

**Table A2:** Summary of mass spectrometry identification and quantitative analysis of modified proteins in the hippocampus subjected to IR injury and IPC.

Spot No.	Protein ID	Protein Name	Gene	Mw (kDa)	pI	IR/CON Fold Change/*p*-Value	IPC/CON Fold Change/*p*-Value
1	CALR	Calreticulin	*Calr*	48.14	4.33	ns	0.33 0.0037 **
2	GFAP	Glial fibrillary acidic protein beta	*Gfap*	38.52	5.35	1.77 0.047 *	2.04 0.021 *
3	STMN1	Stathmin	*Stmn1*	17.28	5.76	ns	0.38 0.023 *
4	UBE2N	Ubiquitin-conjugating enzyme E2	*Ube2n*	17.17	6.13	ns	0.44 0.068
5	1433Z	14-3-3 protein zeta/delta	*Ywhaz*	27.92	4.73	1.85 0.028 *	2.28 0.032 *
6	PRDX6	Peroxiredoxin 6	*Prdx6*	24.86	5.64	ns	0.36 0.0076 **
7	ANXA3	Annexin 3	*Anxa3*	36.57	5.96	ns	1.53 0.0067 **
8	IDH3A	Isocitrate dehydrogenase 3 (NAD+) alpha, mitochondrial	*Idh3a*	40.04	6.47	ns	0.38 0.036 *
9	ATPB	ATP synthase subunit beta, mitochondrial	*Atp5b*	56.32	5.15	ns	2.07 0.033 *
10	SDHA	Succinate dehydrogenase [Q] flavoprotein subunit	*Sdha*	72.60	6.75	0.62 0.029 *	0.49 9 × 10^−4^ ***
11	GRP75	75 kDa glucose-regulated protein	*Hspa9*	74.10	5.97	0.51 0.0042 **	ns
12	ALDR	Aldo-keto reductase family 1 member B1	*Akr1b1*	36.23	6.26	ns	ns
13	IVD	Isovaleryl-CoA dehydrogenase, mitochondrial	*Ivd*	46.86	8.03	ns	ns
14	ENOA	Alpha enolase	*Eno1*	47.44	6.16	3.08 0.031 *	2.23 0.033 *
15	HS71A	Heat shock protein70 kDa protein 1A	*Hspa1a*	70.43	5.61	Spot missing in the control group	
16	PRDX5	Peroxiredoxin 5	*Prdx5*	22.51	8.94	ns	0.54 0.0063 **
17	G3P	Glyceraldehyde-3 phosphate dehydrogenase	*Gapdh*	36.09	8.14	ns	1.86 0.043 *
18	ALDOA	Fructose bisphosphate aldolase A	*Aldoa*	39.78	8.31	ns	0.64 0.049 *
19	GLNA	Glutamine synthetase	*Glul*	42.98	6.64	1.51 0.0018 **	1.56 0.0014 **
20	DHE3	Glutamate dehydrogenase, mitochondrial	*Glud1*	61.72	8.05	ns	ns
21	ZUP1	Zinc finger-containing ubiquitin peptidase 1	*Zup1*	66.99	6.08	1.71 0.050 *	ns
22	DPYL4	Dihydropyrimidinase-related protein 4	*Dpysl4*	61.62	6.3	ns	0.46 0.012 *
23	KPYM	Pyruvate kinase	*Pkm*	58.29	6.63	0.60 0.022 *	ns
24	SYN2	Synapsin 2	*Syn2*	63.70	8.73	ns	0.38 0.015 *
25	ACON	Aconitate hydratase	*Aco2*	86.12	7.87	1.68 0.019 *	ns
26	PIMT	Protein-D-aspartate-O-methyltransferase	*Pcmt1*	24.68	7.14	ns	0.35 0.0099 **
27	KAD1	Adenylate kinase isoenzyme 1	*Ak1*	21.68	7.66	ns	ns
28	VDAC2	Voltage-dependent anion-selective channel protein 2	*Vdac2*	32.35	7.44	ns	0.47 0.024 *
29	ROA2	Heterogenous nuclear ribonucleoproteins A2/B1	*Hnmpa2b1*	37.51	8.94	0.56 0.029 *	0.26 0.026 *

* *p* < 0.05, ** *p* < 0.01, *** *p* < 0.001; significantly different when compared to the control.
